# Oral Hemorrhage in a 3-year-old Boy Caused by Self-Mutilating Behavior

**DOI:** 10.12669/pjms.326.12088

**Published:** 2016

**Authors:** Jenny Lyn Y. Uy, José Florencio F. Lapeña

**Affiliations:** 1 Dr. Jenny Lyn Y. Uy, BS, MD. Department of Otorhinolaryngology, Philippine General Hospital, University of the Philippines Manila, Philippines; 2 Prof. Dr. José Florencio F. Lapeña, Jr., MA, MD. Department of Otorhinolaryngology, College of Medicine, Philippine General Hospital, University of the Philippines Manila, Philippines

**Keywords:** Lesch-Nyhan syndrome, Hypoxanthine-guanine phosphoribosyl transferase 1 deficiency, Oral hemorrhage, Self-mutilation, Self-injurious behavior

## Abstract

A 3-year-old boy referred for persistent tongue bleeding was diagnosed with a rare self-mutilating disease that had also affected his lip and fingers. He underwent multiple odontectomy and partial glossectomy and continues to undergo behavior therapy and on-demand splints and restraints. He has stopped self-biting and has gained appetite and weight. Lesch-Nyhan Syndrome can cause significant morbidity including self-inflicted oral hemorrhage and emergent measures are not easily decided. The long-term management of its neurobehavioral symptoms is problematic and multidisciplinary, and health providers remain challenged to find the best treatment, prolong lifespan and improve quality of life within their respective contexts.

## INTRODUCTION

The oral cavity is involved in chewing, swallowing, speech and respiration. It is also highly vascular, and simple trauma can cause severe bleeding. Because it is intimately related to the upper aerodigestive tract, excessive bleeding can seriously compromise the airway, requiring emergent management. Such bleeding becomes more worrisome when injury is self-inflicted. Self-mutilation is usually associated with psychiatric conditions, but may have an organic cause. We describe such a case.

## CASE REPORT

A 3-year-old boy was referred for urgent evaluation and management of self-inflicted tongue bleeding that could not be controlled by anticoagulants and oral gauze packs. Active bleeding episodes previously resolved medically until the present exacerbation. On review of history, the mother recalled delayed motor development, with inability to hold up the head or sit without support at seven months of age, for which a diagnosis of cerebral palsy was made. There was not much improvement with physical therapy until he started biting his lower lip and fingers at about two years of age. A pediatric neurologist considered a seizure disorder and requested biochemical tests that confirmed Lesch-Nyhan syndrome. The self-mutilating behavior was not controlled with diazepam and allopurinol, with progressive loss of the lower lip and fingertips.

**Fig.1:**
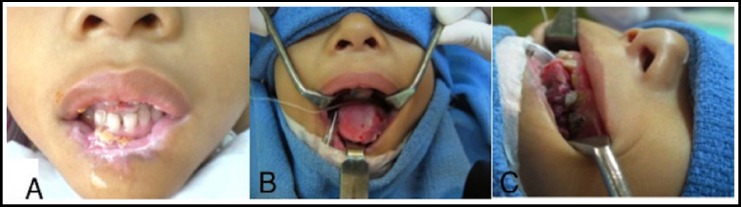
A: Absent central lower lip; B, C: Intraoperative views of macerated anterolateral third of left tongue.

**Fig.2:**
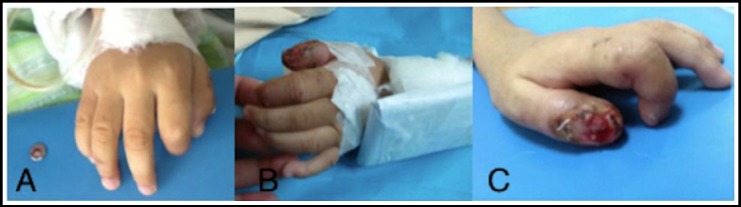
Amputated tips of both index fingers- A: Right hand; B, C: Left hand with eroded distal left thumb.

On examination, the central lower lip was absent, and the anterolateral third of the left tongue was macerated and actively bleeding. (Fig.1 A, B, C) The tips of both index fingers were amputated and the distal left thumb was eroded, with raw and necrotic areas. (Fig.2 A, B, C) Following an earnest conversation, the boy agreed to continue biting on a gauze pad, and to signal when he felt a compulsion to bite his fingers, in order for arm restraints to be secured. He assented, and his mother consented to multiple odontectomy, partial glossectomy and debridement, control of bleeders and tongue repair under general anesthesia.

Post-operatively, his tongue, gums and digits healed uneventfully. Behavior modification was continued, with extinction and positive reinforcement helping gradually control his biting behavior. He was advised regular follow-up while waiting for permanent dentition to erupt, when oral appliances could be fitted, and lip reconstruction planned.

## DISCUSSION

Lesch-Nyhan syndrome (LNS) is a rare X-linked recessive error of metabolism associated with deficient enzymatic activity of hypoxanthine-guanine phosphoribosyl transferase (HPRT).^1^ It was first described by Lesch and Nyhan in two brothers presenting with hematuria, motor development delay, choreoathethosis, and badly mutilated lips and mouth^2^ although Catel and Schimdt previously reported an infant with hyperuricemic encephalopathy who was later shown to have HPRT deficiency.^3^ Although the pathways leading from genotype to phenotype remain unclear,^4^ the characteristic neuropsychiatric manifestations of LNS may be caused by aberrant development of HPRT-deficient dopaminergic neurons.^5^ By mechanisms that still need to be elucidated, this manifests with self-mutilating behavior, including uncontrolled self-biting of the lips, tongue and buccal area that may seriously compromise the airway by causing massive bleeding that may require emergent management.

While there is little room for argument regarding immediate surgical soft-tissue hemostasis (in this case, through partial glossectomy and debridement), the need for odontectomy is a difficult management choice in the face of such compulsive, “severe and recurrent self-injurious behavior,” but it “should not be delayed when biting is severe”.^6^ In our case, the functional and cosmetic consequences of complete extraction far outweighed the benefits of retaining dentition.

Intraoral devices may have been an option, but they are “not free of complications,” and “therapy must be individualized” in the absence of “standardized treatment protocols”.^7^ They may still be utilized for future permanent dentition, following maximal multi-modal therapy including pharmacotherapy, psychosocial counseling and behavioral intervention.

Although their usefulness has been well described, behavioral interventions “have been limited in number and long-term success”.^8^ In our case, earnest communication after establishing rapport was successful in initially modifying self-injurious behavior. Investigative therapies including gabapentin to “improve self-injurious behavior”^1^,^9^ and botulinum toxin masseteric injections^10^ as well as globus pallidus deep-brain stimulation or dopamine replacement therapy “need to prove to be efficacious and safe in the long-term management of these patients”^1^ and (especially the latter) are not easily available or accessible in low- and middle-income countries such as ours.

Indeed, Lesch-Nyhan syndrome can cause significant morbidity including self-inflicted oral hemorrhage, and the necessary emergent measures are not easily decided. The long-term management of its neurobehavioral symptoms is problematic and multidisciplinary, and health providers remain challenged to find the best treatment, prolong lifespan and improve quality of life within their respective contexts.
